# P-409. Reevaluation of Contamination Rates: Discrepancies Between Clinical and Laboratory Assessment

**DOI:** 10.1093/ofid/ofae631.610

**Published:** 2025-01-29

**Authors:** Hiroshi Hamada, Hiroshi Morioka, Mitsutaka Iguchi, Keisuke Oka, Kohei Kanda, Tetsuya yagi, Masaki Okazaki, Atsushi Hashizume

**Affiliations:** Nagoya University Hospital, Nagoya, Japan., nagoya city, Aichi, Japan; Nagoya University Hospital, Nagoya, Japan., nagoya city, Aichi, Japan; Nagoya University Hospital, Nagoya, Japan., nagoya city, Aichi, Japan; Nagoya University Hospital, Nagoya, Japan., nagoya city, Aichi, Japan; Nagoya University Hospital, Nagoya, Japan., nagoya city, Aichi, Japan; Nagoya University Hospital, Nagoya, Japan., nagoya city, Aichi, Japan; Nagoya University Graduate School of Medicine, Nagoya, Japan, nogoya city, Aichi, Japan; Nagoya University Graduate School of Medicine, Nagoya, Japan, nogoya city, Aichi, Japan

## Abstract

**Background:**

Contamination of blood cultures can result in unnecessary testing and the unwarranted use of antimicrobials.Quality control is essential to prevent these outcomes, with the contamination rate serving as a key quality indicator.

Various criteria exist for assessing contamination prevalence. While these criteria enable independent computation of contamination rates by the laboratory, they can differ significantly from clinician judgments. But recent advancements, such as improved blood culture bottles, new disinfectants, the use of mass spectrometers, and the rise in immunosuppressed patients and intravascular device users, may have influenced these divergences.

Table1

**Figure:**
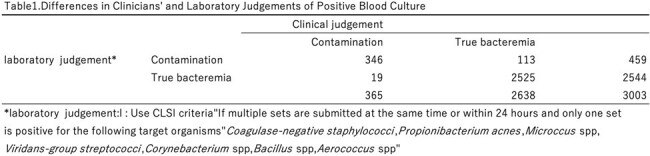

laboratory judgement : Use CLSI criteria"If multiple sets are submitted at the same time or within 24 hours and only one set is positive for the following target organisms"Coagulase-negative staphylococci,Propionibacterium acnes,Microccus spp,Viridans-group streptococci,Corynebacterium spp,Bacillus spp,Aerococcus spp"

**Methods:**

Over a four-year period starting in 2018, we conducted a retrospective cohort study on patients with multiple-set blood cultures submitted at our hospital. This study differentiated contamination from true bacteremia, as defined by the laboratory and as assessed by infectious disease specialists. The definition of contamination by the laboratory followed the criteria of CLSI.

**Figure:**
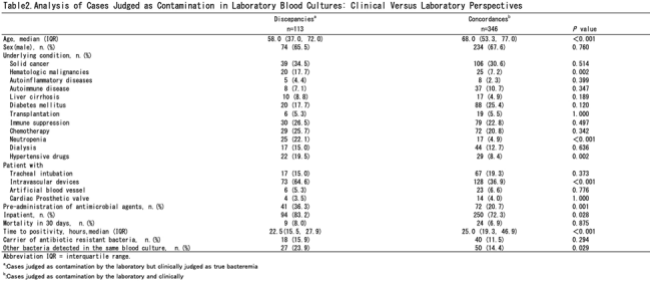

Abbreviation IQR = interquartile range.

a:Cases judged as contamination by the laboratory but clinically judged as true bacteremia

b:Cases judged as contamination by the laboratory and clinically

**Results:**

Of the 23,472 blood culture submissions, 18,851 involved multiple sets. Of these, 3,003 cases tested positive in at least one set. The laboratory identified 459 cases as contamination, while clinicians identified 365 cases. The contamination rates were 2.4% and 1.9%, respectively. Discrepancies were notably higher among patients with haematological malignancies, neutropenia, those with intravascular devices, and when multiple bacteria were concurrently detected, particularly in cases involving *Staphylococcus epidermidis*.

**Figure:**
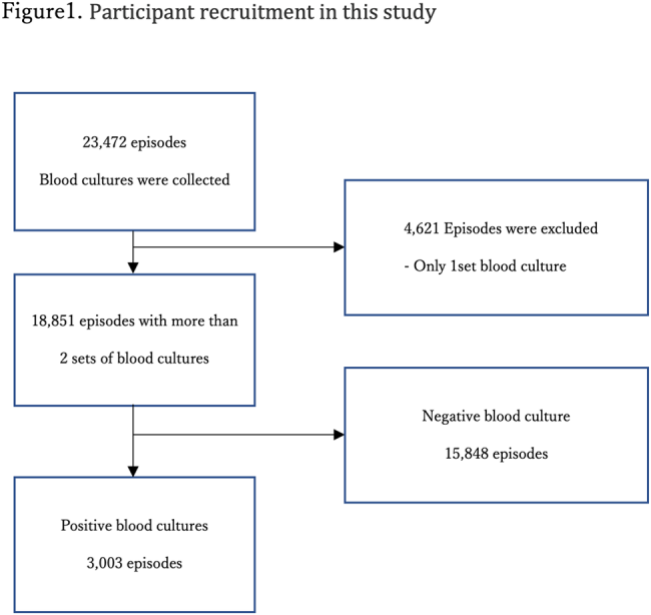

**Conclusion:**

It has been reported that there is little difference between the judgment of contamination between the judgment of the laboratories and the clinicians, however,

in the present study a large discrepancy in contamination rates was observed.

This may be related to patients’background in our hospital where a large number of patients with haematological malignancies and neutropenia, and patients with intravascular devices were hospitalized

,and the clinical side tended to treat such patients as having true bacteremia.

The possibility of a divergence in contamination rates as a quality indicator should be considered in such facilities.

**Figure:**
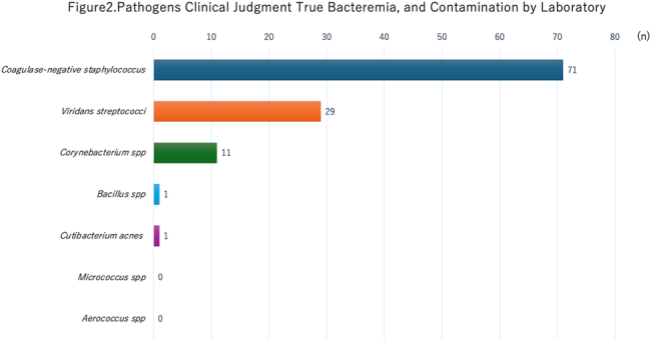

**Disclosures:**

**Hiroshi Morioka, MD-PhD**, SHIMADZU CORPORATION: Advisor/Consultant **Mitsutaka Iguchi, n/a**, SHIMADZU CORPORATION: Advisor/Consultant **Keisuke Oka, MD-PhD**, SHIMADZU CORPORATION: Advisor/Consultant **Tetsuya yagi, MD-PhD**, SHIMADZU CORPORATION: Advisor/Consultant

